# Supplementary material for: Integrated Genomic and Phenotypic Analysis of Bacillus safensis LG01 Highlights Its Prospects in Biotechnology and Biocontrol
Source: Microorganisms. 2025 Nov 15;13(11):2605. doi: 10.3390/microorganisms13112605 (PMC12654287; doi:10.3390/microorganisms13112605)
Supplement: Supplementary file 1 [file microorganisms-13-02605-s001.zip › microorganisms-3925433-supplementary.pdf]

Supplementary Information

Figure S1

Genomic islands identified in *B. safensis* LG01.

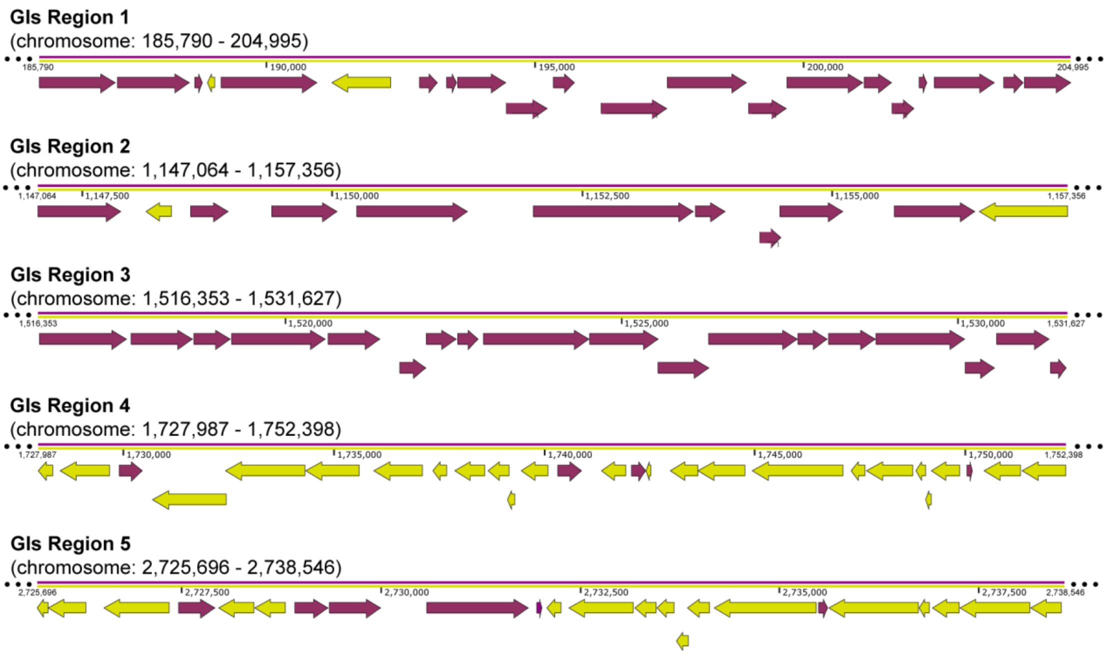

Figure S2

Prophage regions identified in *B. safensis* LG01.

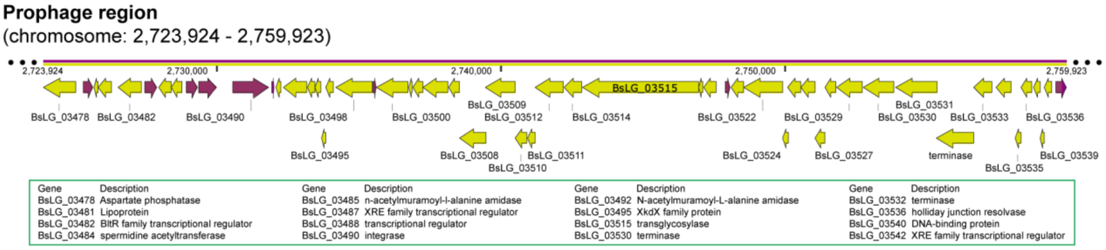

Figure S3

The result of classification statistics of KEGG pathway annotation of *B. safensis* LG01.

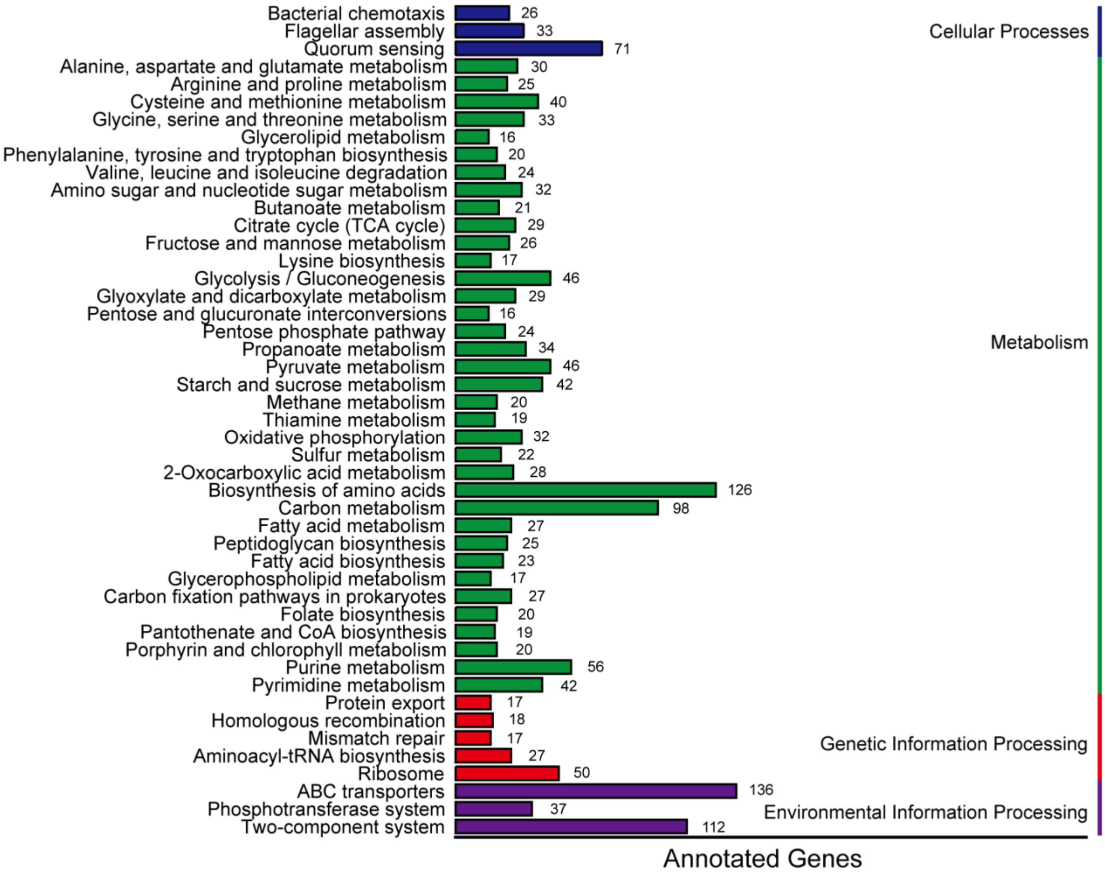

Figure S4

Synteny map of four sub clusters of *B. safensis* strains.

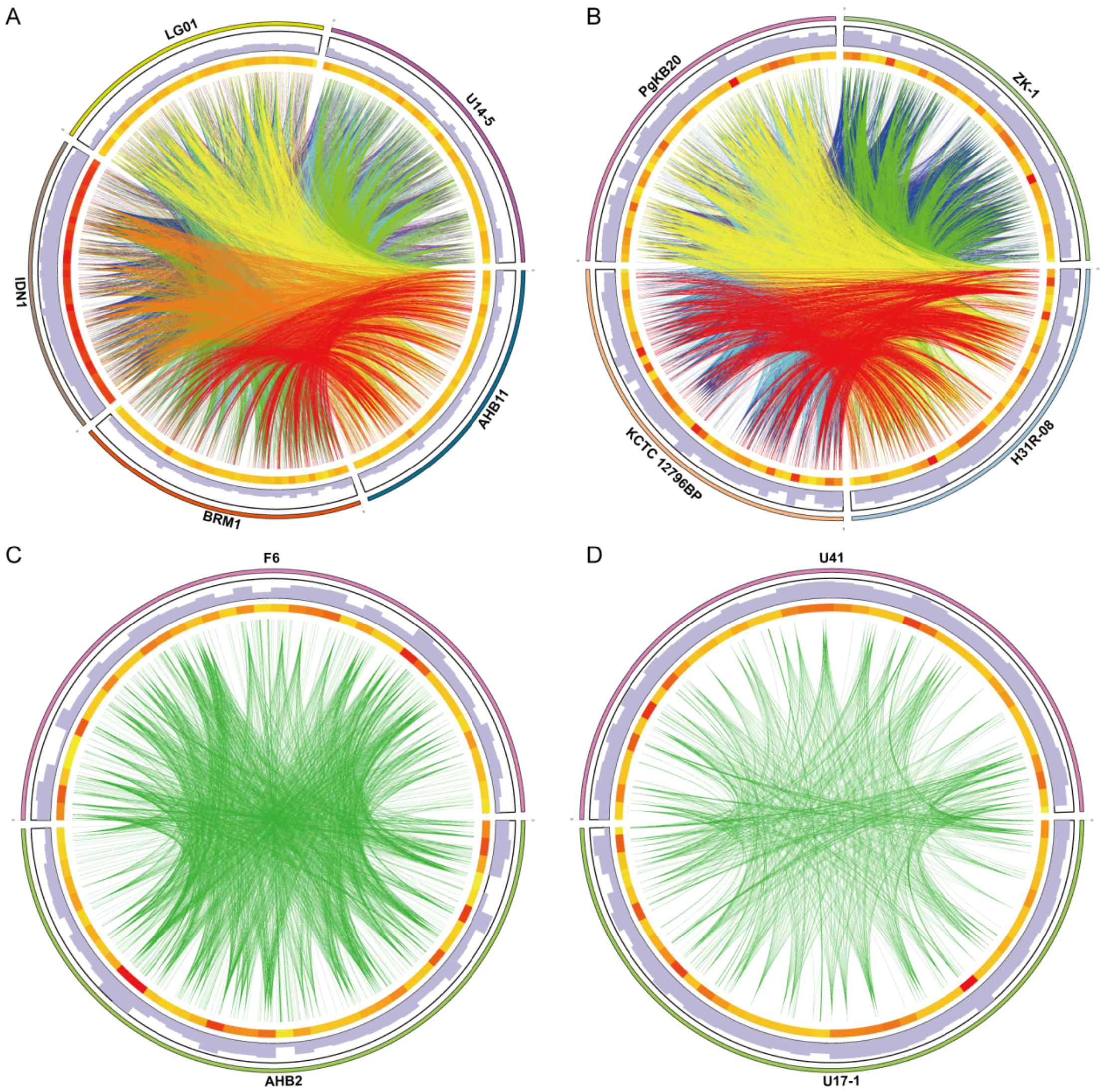

(A), (B), (C), and (D) represent the genomic comparisons of *B. safensis* strains belonging to Class I, Class II, Class III, and Class IV, respectively. The outermost circle is labeled with the genome name; the second and third circles are gene frequencies; the inner region with different colored lines indicates collinearity between genomes.

Table S1

Results of the whole genome sequence comparison.

Strain	Genome size (bp)	GC content (%)	Protein-coding gene	tRNA number	rRNA number	Isolation source	Accession number	Country
LG01	3,664,251	41.73	3,836	81	24	Laboratory environment	CP109651.1	China
U41	3,735,181	41.50	3,721	81	24	Lake Untersee	CP015610	Antarctica
AHB11	3,697,357	41.79	3,668	81	24	Beehive	CP097374	South Sudan
IDN1	3,794,970	41.40	7,225	78	24	food (commercially available natto)	AP021906	Indonesia
U14-5	4,070,488	41.40	4,138	83	24	Lake Untersee	CP015607	Antarctica
KCTC 12796BP	3,935,874	41.36	3,907	81	24	Marine sponge	CP018197	South Korea
AHB2	3,785,324	41.70	3,762	80	24	Beehive	CP097373	South Sudan
F6	3,782,236	41.50	3,699	81	24	Chicken feces	CP069061	Belarus
H31R-08	3,745,092	41.70	3,657	81	24	Soil	CP090354	South Korea
PgKB20	3,786,744	41.70	3,698	81	24	Ginseng seedlings	CP043404	South Korea
U17-1	3,741,804	41.50	3,724	90	27	Lake Untersee	CP015611	Antarctica
BRM1	3,740,842	41.80	3,717	91	24	Mangrove soil	CP018100	Brazil
ZK-1	3,735,476	41.70	3,641	81	24	Soil	CP095759	China

Table S2

Comparison of Average Nucleotide Identity (ANI) among LG01 and Other Strains.

	strain	GenBank	ANI (%)
LG01	AHB11	GCA_023716825.1	98.52
	IDN1	GCA_009936195.2	98.17
	BRM1	GCA_002077215.1	98.87
	U14-5	GCA_001938665.1	97.69
	ZK-1	GCA_023093895.1	97.61
	PgKB20	GCA_008244765.1	97.61
	KCTC 12796BP	GCA_001895885.1	97.64
	H31R-08	GCA_021398835.1	97.60
	AHB2	GCA_023716805.1	96.58
	F6	GCA_016803835.1	96.66
	U17-1	GCA_001938705.1	96.35
	U41	GCA_001938685.1	93.36

Table S3

Antibiotic resistance genes identified in *B. safensis* LG01.

Gene ID	Position	Resistance profile	Resistance type	Description
BsLG_02251	1,789,315 - 1,790,835	MFS transporter	mdr	Major facilitator superfamily (MFS) transporter facilitates the transport across cytoplasmic or internal membranes of one or more from a variety of substrates including ions, sugar phosphates, drugs, neurotransmitters, nucleosides, amino acids, and peptides
BsLG_03549	2,762,524 - 2,763,402	bacitracin	baca	Undecaprenyl pyrophosphate phosphatase, which consists in the sequestration of Undecaprenyl pyrophosphate.

Table S4

Antimicrobial Susceptibility of *B. safensi* LG01.

Name	Abbreviation	Concentration (μg/mL)	Inhibition zone dia. (mm)
Ciprofloxacin	CFX	5	37.8 ± 1.0
Ceftriaxone	CTR	30	35.8 ± 0.8
Trimethoprim Sulfamethoxazole	T/S	25	32.6 ± 0.8
Erythromycin	ERM	15	33.7 ± 0.7
Penicillin	PEN	6	31.0 ± 0.5
Chloramphenicol	CLM	30	31.5 ± 0.9
Gentamicin	GEN	10	24.6 ± 1.1
Ampicillin	AMP	10	22.3 ± 0.5
Tetracycline	TET	30	21.9 ± 0.4
Lincomycin	LIN	2	0.0

Table S5

Representative genes of *B. safensis* LG01 probably involved in host-bacteria interactions.

#	Gene	Position	Protein description
**Putative secreted proteins**	BsLG_00300	255,230 - 256,843	Peptidase S8
	BsLG_00711	614,163 - 615,092	Peptidase S8
	BsLG_00941	798,724 - 799,476	Cell surface protein with WxL domain
	BsLG_01213	1,001,217 - 1,002,362	Serine alkaline protease (subtilisin E)
	BsLG_01459	1,181,344 - 1,183,092	Gamma-glutamyltranspeptidase
	BsLG_01808	1,426,668 - 1,430,984	Peptidase S8
	BsLG_02332	1,868,343 - 1,869,050	1,4-beta-xylanase
	BsLG_02651	2,098,280 - 2,099,410	Subtilisin
	BsLG_04032	3,121,336 - 3,122,598	Peptidase M23
	BsLG_04104	3,183,791 - 3,186,421	Beta-N-acetylhexosaminidase
	BsLG_04364	3,378,319 - 3,380,745	Peptidase S8
	BsLG_04446	3,434,333 - 3,435,361	Pectate lyase
**Quorum sensing**	BsLG_03440	2,691,479 - 2,691,952	Quorum sensing signal autoinducer-2 LuxS
	BsLG_04097	3,174,619 - 3,176,985	Minor teichoic acid biosynthesis protein
	BsLG_04094	3,171,090 - 3,171,971	Minor wall teichoic acid biosynthetic enzyme GtaB
	BsLG_02744	2,164,044 - 2,164,847	The master regulator Spo0A
	BsLG_04580	3,551,895 - 3,553,073	Rap-phr extracellular signaling
	BsLG_02742	2,162,487 - 2,163,587	Rap-phr extracellular signaling
	BsLG_04169	3,240,050 - 3,241,105	Rap-phr extracellular signaling
	BsLG_01426	1,162,130 - 1,163,263	Rap-phr extracellular signaling
	BsLG_02061	1,642,583 - 1,643,731	Rap-phr extracellular signaling
	BsLG_04411	3,413,290 - 3,414,375	Rap-phr extracellular signaling
	BsLG_02106	1,677,753 - 1,678,907	Rap-phr extracellular signaling

Table S6

List of various bioactive metabolites synthesized by *B. safensis* LG01 and the closely related species.

Bacillus Species	Bioactive Metabolite
B. safensis LG01	Amylocyclicin, Fengycin, Surfactin, Bacillomycin-D, Bacillibactin, Bacillaene, Difficidin, Macrolactin, Plantazolicin, Bacilysin,
B. subtilis	Bacillibactin, Bacillaene, Bacilysin, Difficidin, Bacitracin, Fengycin, Locillomycin, Subtilosin, Surfactin
B. amyloliquefaciens subsp. amyloliquefaciens	Bacillibactin, Bacillaene, Bacillomycin-D, Bacilysin, Fengycin, Surfactin
B. siamensis	Bacillomycin-D, Bacillaene, Difficidin, Fengycin, Surfactin
B. licheniformis	Bacitracin, Lichenysin, Lichenin
B. pumilus	Amicoumacin, Bacilysin, Bacircine, Pumilacidin

Reference

Rabbee MF, Ali MS, Choi J, Hwang BS, Jeong SC, Baek KH. *Bacillus velezensis:* A Valuable Member of Bioactive Molecules within Plant Microbiomes. Molecules. 2019 Mar 16;24(6):1046. doi: 10.3390/molecules24061046.
